# Supplementary material for: Characterizing the Cell-Free Transcriptome in a Humanized Diffuse Large B-Cell Lymphoma Patient-Derived Tumor Xenograft Model for RNA-Based Liquid Biopsy in a Preclinical Setting
Source: Int J Mol Sci. 2024 Sep 16;25(18):9982. doi: 10.3390/ijms25189982 (PMC11432451; doi:10.3390/ijms25189982)
Supplement: Supplementary file 1 [file ijms-25-09982-s001.zip › ijms-3191957-supplementary.pdf]

Supplementary figures

captions

Supplementary Figure 1: Change in baseline weights from the start of tumor inoculation in HIS (top) and non-HIS (bottom) NSG mice. For the grouped analyses, sacrificed mice kept their last weight to avoid inappropriate improvement of group averages. Vertical lines indicate IV treatments (CHOP only given once). The horizontal line represents our chosen humane endpoint for sacrifice. Error bars represent the SEM. HIS: human Immune System; SEM: standard Error of the Mean.

Supplementary Figure 2: Endogenous human (left) and murine (right) cfRNA concentrations in HIS and non-HIS PDTX NSG mice treated with PBS, R-CHOP, and hu-CD20-FC-AFN-CHOP. Significant p-values are shown. CfRNA: cell-free RNA; HIS: human immune system; NSG: NOD-*scid* IL2Rgnull; PBS: phosphate-buffered saline; PDTX: patient-derived tumor xenograft. Pre-PDTX: before xenograft implantation; Pre-Tx: pretreatment; Post-Tx: posttreatment.

Supplementary Figure 3: Spearman rank correlation between tumor volume (mm3) and the number of unique human (left) and murine (right) genes within the non-HIS xenografted PBS-treated NSG mice. Log-transformed values are shown. HIS: human immune system; NSG: NOD-*scid* IL2Rgnul; PBS: phosphate-buffered saline; PDTX: patient derived tumor xenograft; R: spearman correlation coefficient.

Supplementary Figure 4: Human cell-free RNA concentration (A) and fraction of human counts (B) in PBS-treated NSG mice that were only humanized (HIS non-PDTX), only xenografted (non-HIS PDTX), or both (HIS PDTX). HIS: humanized; NSG: NOD-*scid* IL2Rgnul; PDTX: patient-derived tumor xenograft.

Supplementary Figure 5: Fraction of human-derived on total cfRNA count in HIS (left) and non-HIS (right) PDTX engrafted NSG mice at the different timepoints of pre-engraftment, pretreatment, after treatment with R-CHOP, hu-CD20-FC-AFN-CHOPor PBS, respectively, and at sacrifice. HIS: human immune system; NSG: NOD-*scid* IL2Rgnull; PBS: phosphate-buffered saline; PDTX: patient derived tumor xenograft; pre-tx: pretreatment; post-tx: posttreatment.

Supplementary Figure 6: Volcano plot depicting the differentially abundant human (A) and murine (B) genes between PBS-treated versus immunochemotherapy-treated PDTX non-HIS mice, between PBS-treated versus immunochemotherapy-treated PDTX HIS mice, and between PBS-treated PDTX versus non-PDTX HIS mice at late stage disease (cutoff q-value =<0.05 and |log2FC|>=1). HIS: human immune system; Log2FC: log2 fold change; PBS:phosphate-buffered saline; PDTX: patient derived tumor xenograft.

Supplementary Figure 7: Dynamic abundance trajectories of *RELN*, *RNVU1-19*, *ABCC6*, *PELP1*, *GOLGA6L24* and *ACTB* in immunochemotherapy- and PBS-treated HIS and non-HIS PDTX NSG mice. Significant p-values are shown. HIS: human immune system; PBS:phosphate-buffered saline; PDTX: patient derived tumor xenograft. NSG: NOD-*scid* IL2Rgnull.

Supplementary Figure 8: Functional annotation and enrichment analysis in PBS-treated PDTX HIS mice. (A) Enrichment dot plot of the term Genetic Ontology. The graph presents the top 10 enriched ontologies for each of the instance terms (biological process, cellular component, and molecular function) with adjusted p-value < 0.05. The X-axis presents the number of genes that enrich the ontology term, and the point size is proportional to this number. (B) Cnetplot depicting the linkages of genes and enriched concepts based on the Network of Cancer Genes database. The color of the circles represents the log2FC. (C) Treeplot showing hierarchical clustering of enriched pathways. The point size is proportional to the number of genes within a pathway. HIS: human immune system. PBS: phosphate-buffered saline; PDTX: patient-derived tumor xenograft.

Supplementary Figure 9: ROC curve showing the performance of the 8-gene signature (AUC 0.92; 95% CI: 0.77-1) to classify PDTX plasma samples by response to therapy at sacrifice timepoint (A). ROC curve showing the performance of the 8-gene signature (AUC 0.84; 95% CI: 0.63-1) to separate FFPE samples of DLBCL patients from healthy lymph node tissue (B). The gene signature was computed as the average normalized abundance value of following genes: *PHF14, PELP1, RPL7L1, HPRT1, RELN, RNU1-75P, RNU5A-8P, RNVU1-19*. AUC: Area under curve. ROC: Receiver Operating Characteristic.

Supplementary Figure 10 (part 1): Normalized counts of the genes included in the signature between the FFPE tissue of DLBCL patients and healthy controls. The DLBCL patient from whom the PDTX is derived is depicted in green. (A) Longitudinal abundance of these genes within the blood plasma of the patient from whom the PDTX is derived (B). CR: complete remission; DLBCL: diffuse large B-cell lymphoma. FFPE: formalin-fixed paraffin embedded; PD: progressive disease; PDTX: patient derived tumor xenograft.

Supplementary Figure 10 (part 2): Normalized counts of the genes included in the signature between the FFPE tissue of DLBCL patients and healthy controls. The DLBCL patient from whom the PDTX is derived is depicted in green. (A) Longitudinal abundance of these genes within the blood plasma of the patient from whom the PDTX is derived (B). CR: complete remission;

DLBCL: diffuse large B-cell lymphoma. FFPE: formalin-fixed paraffin embedded; PD: progressive disease; PDTX: patient derived tumor xenograft.

Supplementary Figure 11: Cumulative abundance of genes present (green) or absent (red) in the cfRNA of non-HIS PDTX PBS-treated samples within the diagnostic FFPE (A) and plasma (B) sample of the patient from which the xenograft is derived (two-sample Kolmogorov–Smirnov test, P < 0.001). Cumulative abundance of genes present (green) or absent (red) in the cfRNA of the patient's plasma within the matched diagnostic FFPE sample (C) of the patient from which the xenograft is derived (two-sample Kolmogorov–Smirnov test, P < 0.001). CfRNA: cell-free RNA; FFPE: formalin-fixed paraffin-embedded; HIS: human immune system; PDTX: patient derived tumor xenograft.

Supplementary Figure 12: Spearman correlation between the abundances of all genes in the diagnostic FFPE compared to the plasma sample of the patient from whom the PDTX is derived (left) and for the genes included in the tumor-specific signature of the PDTX model (right). FFPE: formalin-fixed paraffin-embedded; PDTX: patient-derived tumor xenograft; R: spearman correlation coefficient.

Supplementary Figure 13: Human gene overlap analysis between matched early and late stage PDTX longitudinal samples derived from the same PBS-treated non-HIS (A) and HIS (B) NSG mouse. HIS: human immune system; JI: Jaccard Index; NSG: NOD-*scid* IL2Rgnull; OR: odds ratio; PDTX: patient-derived tumor xenograft.

Supplementary Figure 14: Overlap analysis of unique murine genes between individual mice within the non-HIS PDTX (A), HIS non-PDTX (B), and HIS PDTX groups (C), respectively. Within each group, the number of murine genes shared between the different mice (M1-M8) is depicted. HIS: humanized; PDTX: patient derived tumor xenograft.

Supplementary Figure 15: Flowcytometric evaluation of blood at different timepoints in HIS NSG mice. HIS: human immune system; NSG: NOD-*scid* IL2Rgnull.

Supplementary Figure 16: The fraction of huCD45+ cells on total hu- and muCD45+ positive cells at different timepoints during xenograft implantation and treatment as determined using flowcytometric evaluation of blood. Significant p-values are shown. huCD45+: human CD45+ cells; muCD45+: murine CD45+ cells; PDTX: patient-derived tumor xenograft; pre-tx: pretreatment; post-tx:posttreatment.

Supplementary Figure 17: Flowcytometric evaluation of blood, bone marrow and spleen at sacrifice. The panels show the differences in immune cell composition by organ, and by treatment at sacrifice timepoint. *=P≤0.05;**=P≤0.01;***=P≤0.001. HIS: human immune system; PDTX: patient-derived tumor xenograft.

Supplementary Figure 18: Computational bulk deconvolution results on human cfRNA derived from the diagnostic FFPE (A) and blood plasma sample (B) of the patient from which the PDTX was derived. CfRNA: cell-free RNA; FFPE: formalin-fixed paraffin-embedded; PDTX: patient-derived tumor xenograft.

Supplementary Figure 19: Demographics, clinical characteristics, first-line treatments, and outcomes of DLBCL patients of which the diagnostic FFPE sample was included. Percentages and standard deviations are written in parentheses. COO: cell-of-origin; CR: complete remission; DEL: double-expressor lymphoma; DLBCL NOS: diffuse large B-cell lymphoma not otherwise specified, GCB: germinal center B-cell type; LDH: serum lactate dehydrogenase; NCCN-IPI National Comprehensive Cancer Network International Prognostic Index score; R/R: refractory/relapsed disease.

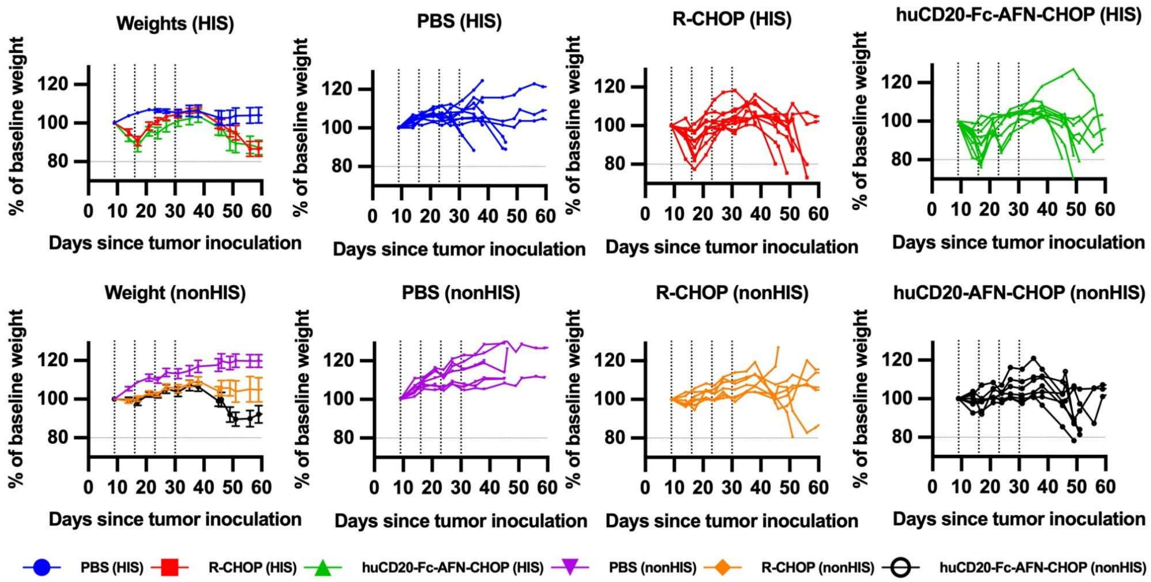

Supplementary Figure S1: Change in baseline weights from the start of tumor inoculation in HIS (top) and non-HIS (bottom) NSG mice. For the grouped analyses, sacrificed mice kept their last weight to avoid inappropriate improvement of group averages. Vertical lines indicate IV treatments (CHOP only given once). The horizontal line represents our chosen humane endpoint for sacrifice. Error bars represent the SEM. HIS: human Immune System; SEM: standard Error of the Mean.

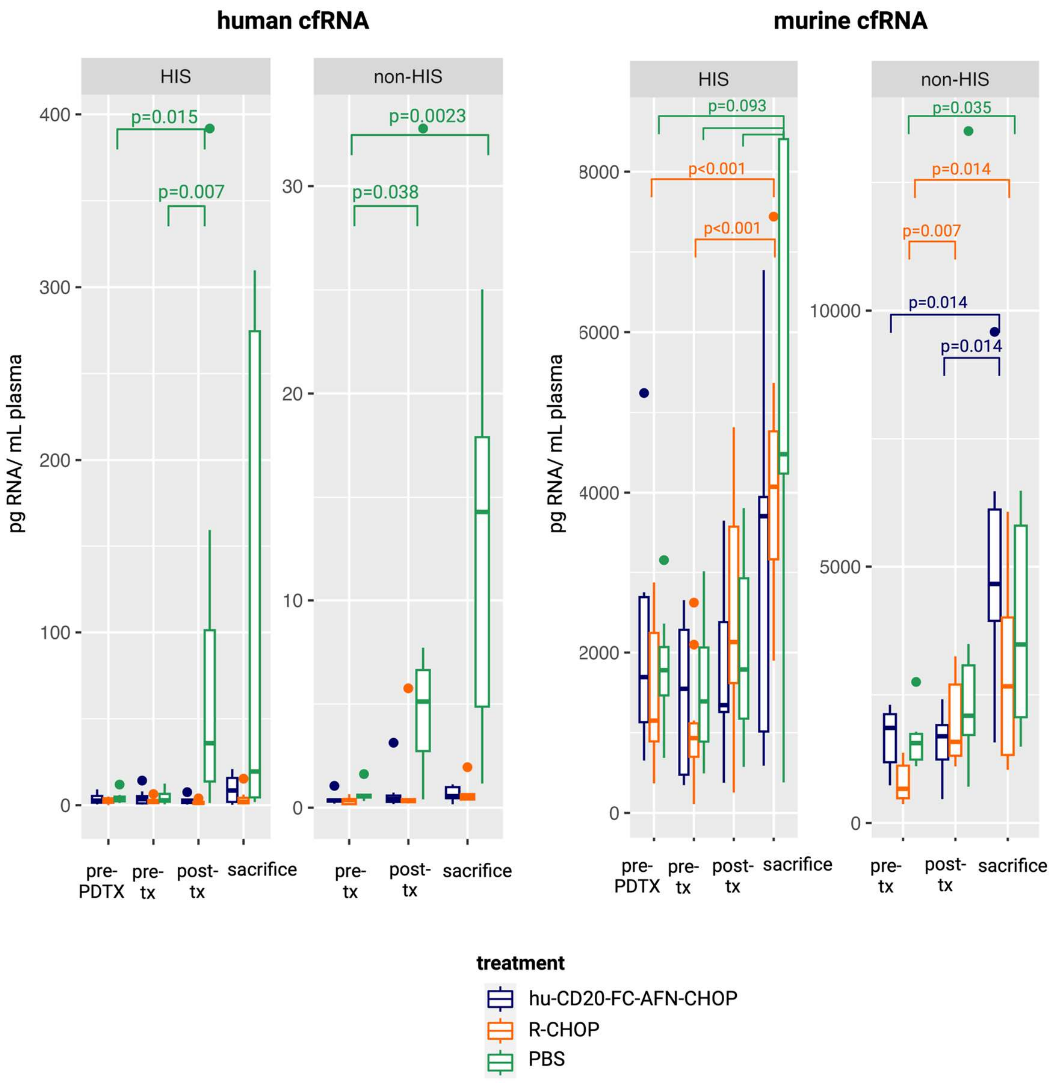

Supplementary Figure S2: Endogenous human (left) and murine (right) cfRNA concentrations in HIS and non-HIS PDTX NSG mice treated with PBS, R-CHOP, and hu-CD20-FC-AFN-CHOP. Significant p-values are shown. CfRNA: cell-free RNA; HIS: human immune system; NSG: NOD-*scid* IL2Rgnull; PBS: phosphate-buffered saline; PDTX: patient-derived tumor xenograft. Pre-PDTX: before xenograft implantation; Pre-Tx: pretreatment; Post-Tx: posttreatment.

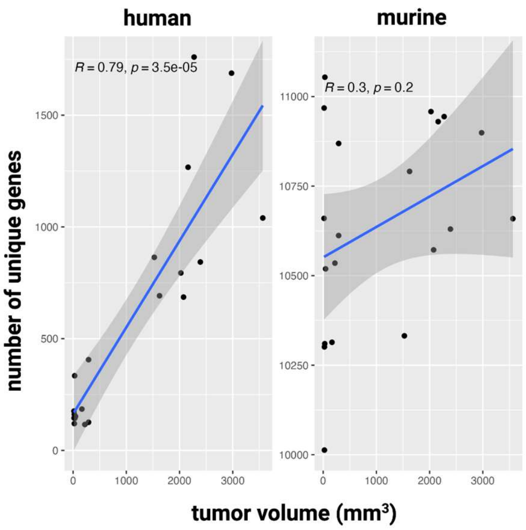

Supplementary Figure S3: Spearman rank correlation between tumor volume (mm3) and the number of unique human (left) and murine (right) genes within the non-HIS xenografted PBS-treated NSG mice. Log-transformed values are shown. HIS: human immune system; NSG: NOD-*scid* IL2Rgnul; PBS: phosphate-buffered saline; PDTX: patient derived tumor xenograft; R: spearman correlation coefficient.

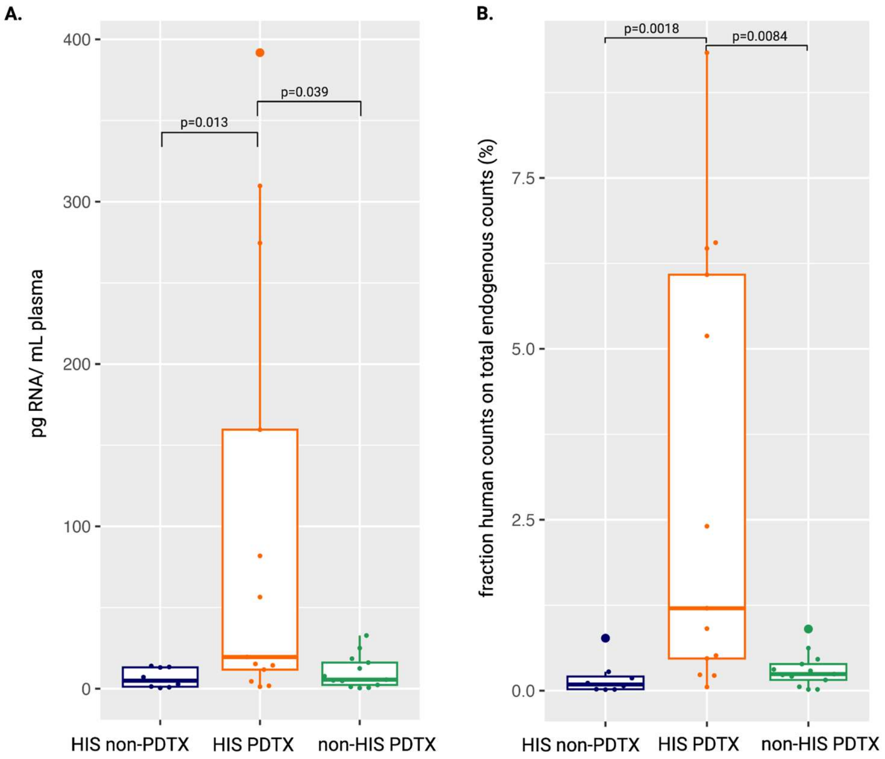

Supplementary Figure S4: Human cell-free RNA concentration (A) and fraction of human counts (B) in PBS-treated NSG mice that were only humanized (HIS non-PDTX), only xenografted (non-HIS PDTX), or both (HIS PDTX). HIS: humanized; NSG: NOD-*scid* IL2Rgnul; PDTX: patient-derived tumor xenograft.

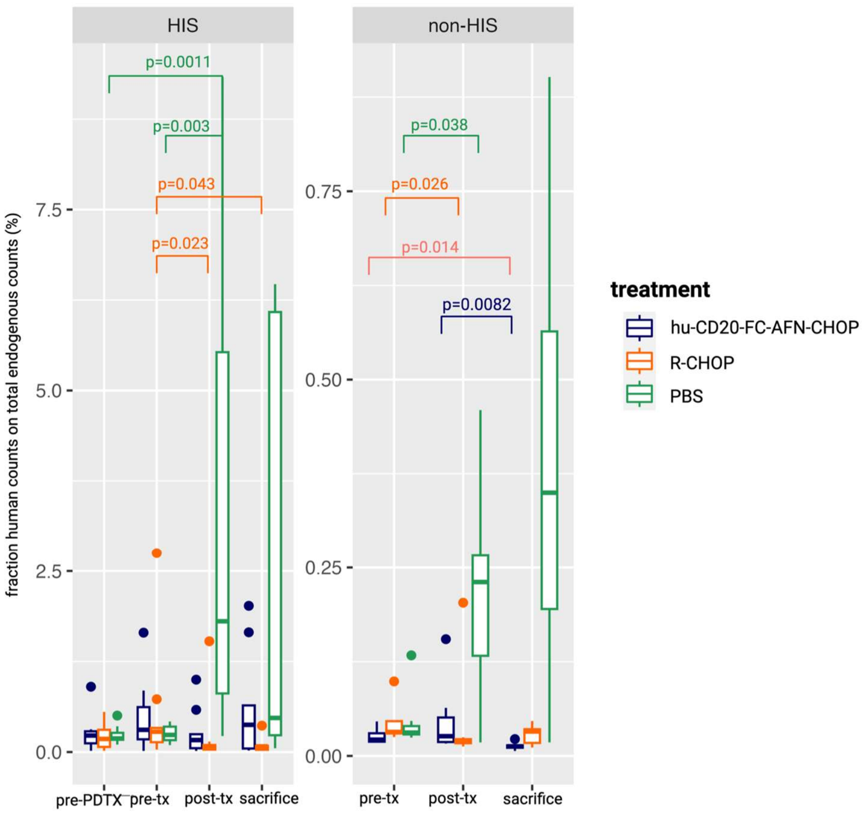

Supplementary Figure S5: Fraction of human-derived on total cfRNA count in HIS (left) and non-HIS (right) PDTX engrafted NSG mice at the different timepoints of pre-engraftment, pretreatment, after treatment with R-CHOP, hu-CD20-FC-AFN-CHOPor PBS, respectively, and at sacrifice. HIS: human immune system; NSG: NOD-*scid* IL2Rgnull; PBS: phosphate-buffered saline; PDTX: patient derived tumor xenograft; pre-tx: pretreatment; post-tx: posttreatment.

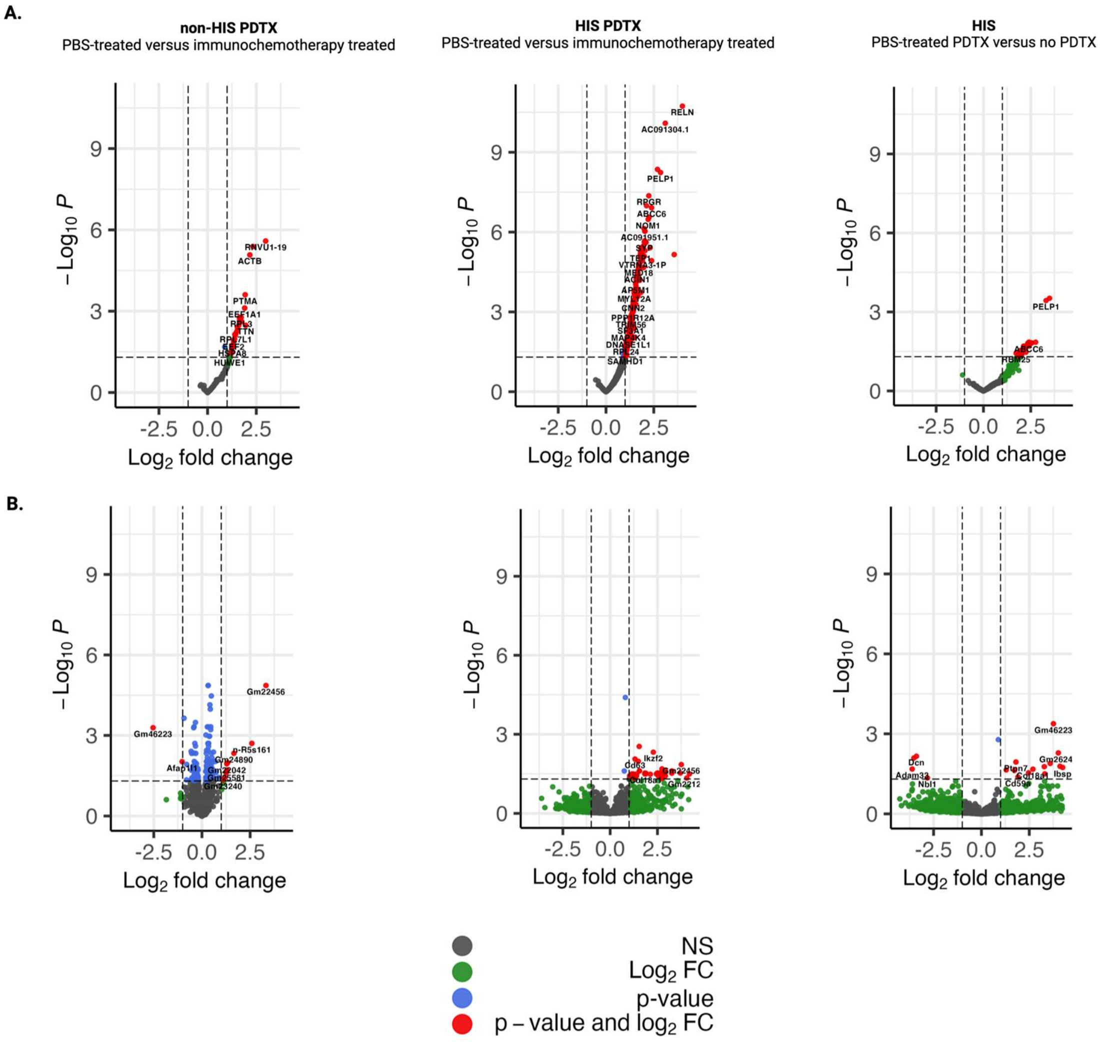

Supplementary Figure S6: Volcano plot depicting the differentially abundant human (A) and murine (B) genes between PBS-treated versus immunochemotherapy-treated PDTX non-HIS mice, between PBS-treated versus immunochemotherapy-treated PDTX HIS mice, and between PBS-treated PDTX versus non-PDTX HIS mice at late stage disease (cutoff q-value =<0.05 and |log2FC|>=1). HIS: human immune system; Log2FC: log2 fold change; PBS:phosphate-buffered saline; PDTX: patient derived tumor xenograft.

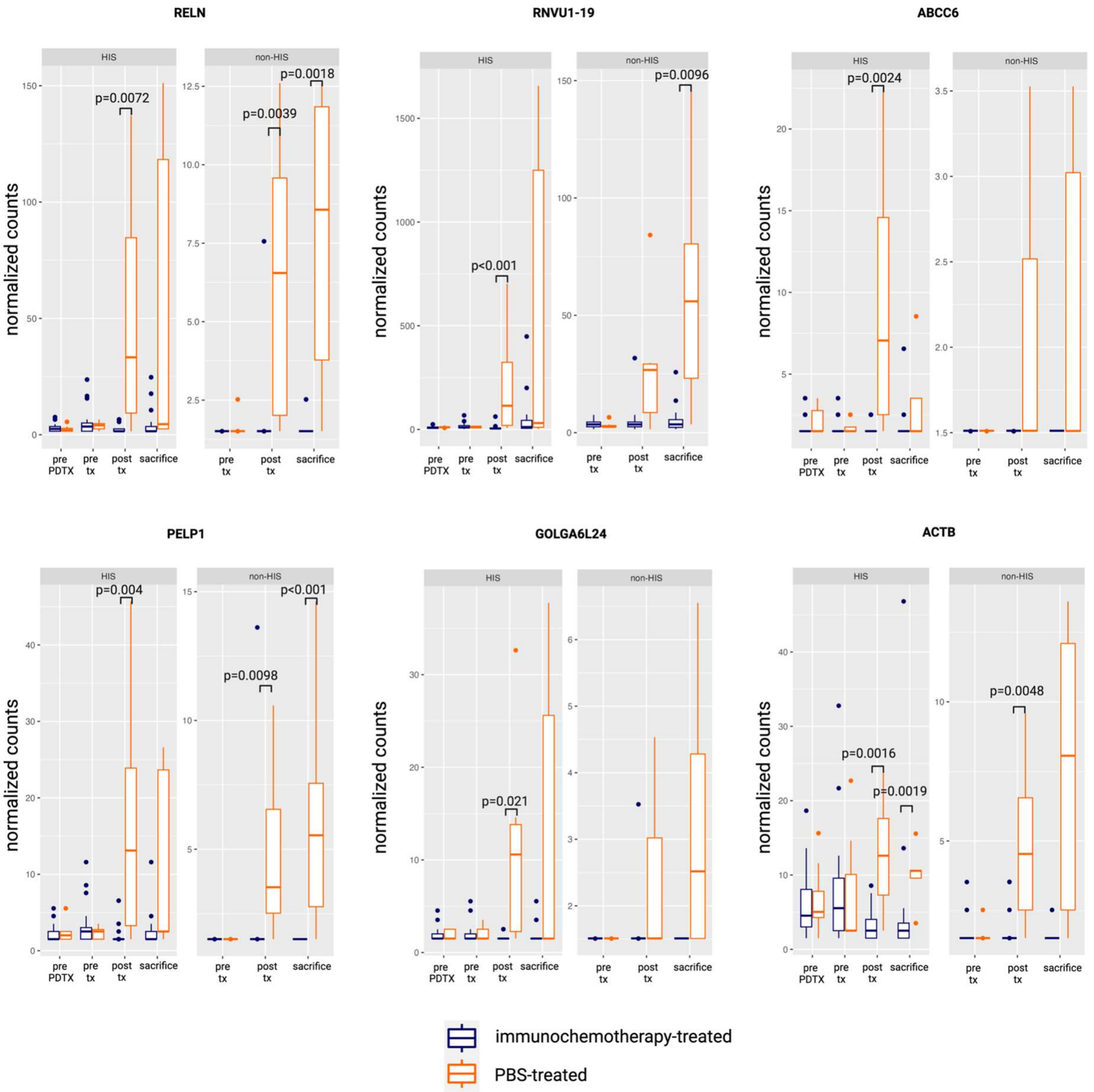

Supplementary Figure S7: Dynamic abundance trajectories of *RELN*, *RNVU1-19*, *ABCC6*, *PELP1*, *GOLGA6L24* and *ACTB* in immunochemotherapy- and PBS-treated HIS and non-HIS PDTX NSG mice. Significant p-values are shown. HIS: human immune system; PBS:phosphate-buffered saline; PDTX: patient derived tumor xenograft. NSG: NOD-*scid* IL2Rgnull.

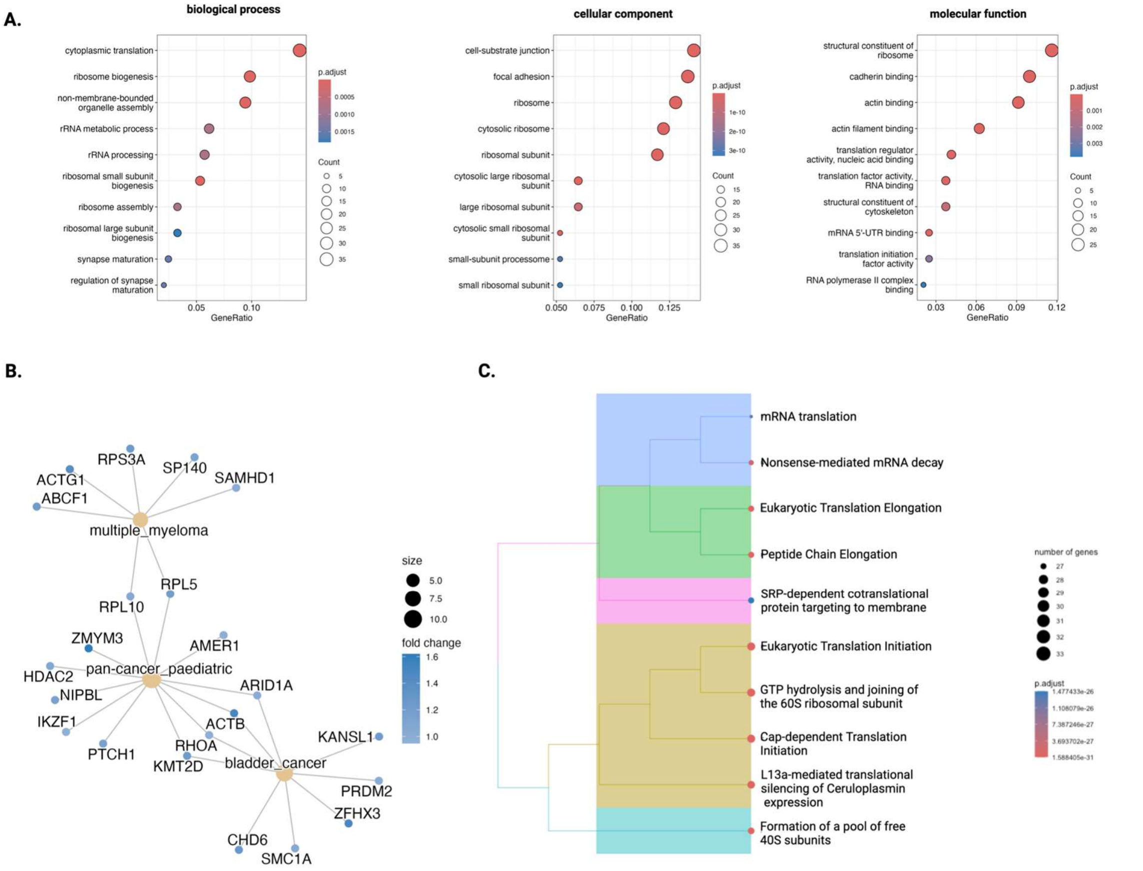

Supplementary Figure S8: Functional annotation and enrichment analysis in PBS-treated PDTX HIS mice. (A) Enrichment dot plot of the term Genetic Ontology. The graph presents the top 10 enriched ontologies for each of the instance terms (biological process, cellular component, and molecular function) with adjusted p-value < 0.05. The X-axis presents the number of genes that enrich the ontology term, and the point size is proportional to this number. (B) Cnetplot depicting the linkages of genes and enriched concepts based on the Network of Cancer Genes database. The color of the circles represents the log2FC. (C) Treeplot showing hierarchical clustering of enriched pathways. The point size is proportional to the number of genes within a pathway. HIS: human immune system. PBS: phosphate-buffered saline; PDTX: patient-derived tumor xenograft.

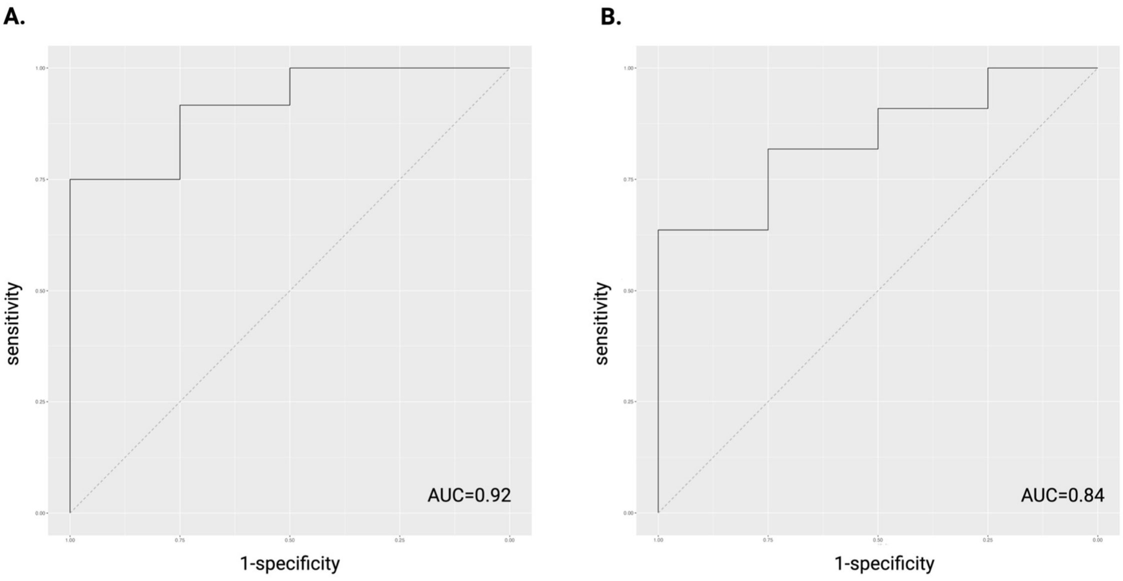

Supplementary Figure S9: ROC curve showing the performance of the 8-gene signature (AUC 0.92; 95% CI: 0.77-1) to classify PDTX plasma samples by response to therapy at sacrifice timepoint (A). ROC curve showing the performance of the 8-gene signature (AUC 0.84; 95% CI: 0.63-1) to separate FFPE samples of DLBCL patients from healthy lymph node tissue (B). The gene signature was computed as the average normalized abundance value of following genes: *PHF14, PELP1, RPL7L1, HPRT1, RELN, RNU1-75P, RNU5A-8P, RNVU1-19*. AUC: Area under curve. ROC: Receiver Operating Characteristic.

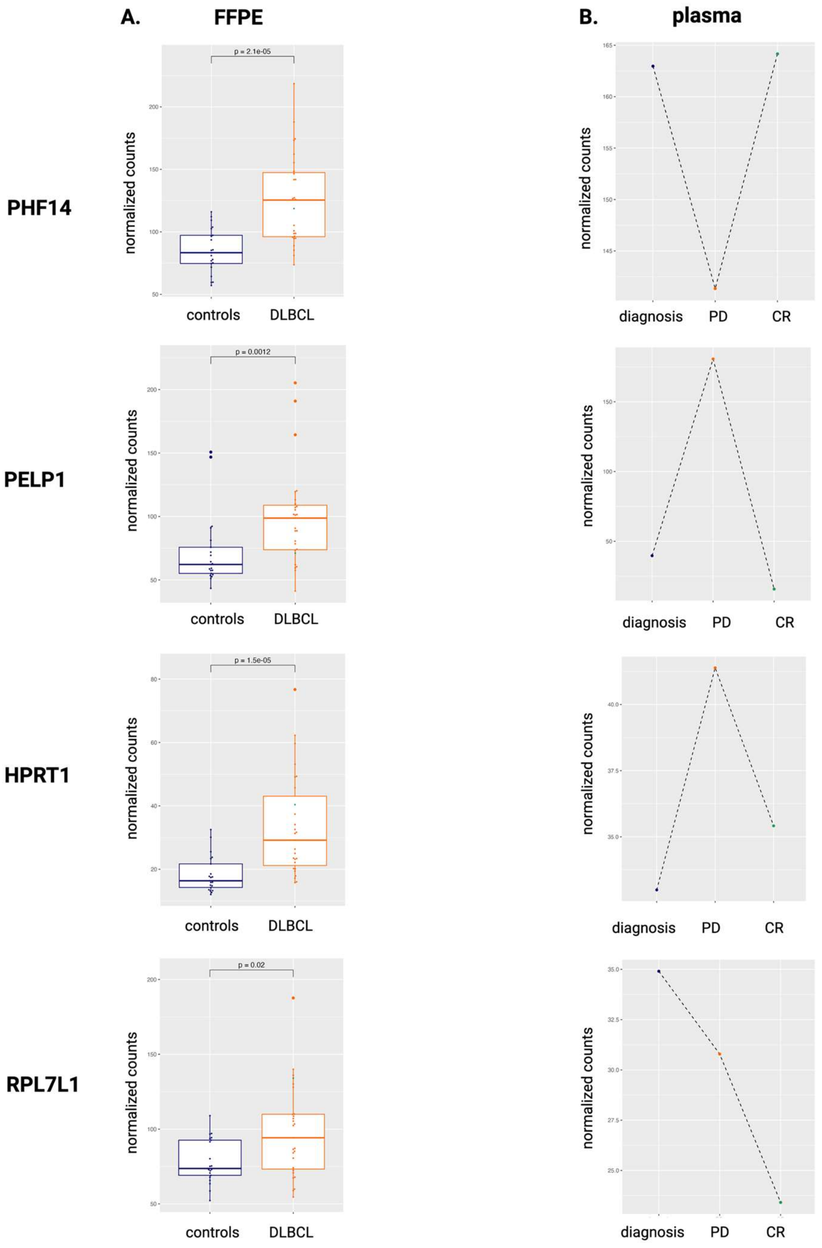

Supplementary Figure S10 (part 1): Normalized counts of the genes included in the signature between the FFPE tissue of DLBCL patients (n=27) and healthy controls (n=21). The DLBCL patient from whom the PDTX is derived is depicted in green. (A) Longitudinal abundance of these genes within the blood plasma of the patient from whom the PDTX is derived (B). CR: complete remission; DLBCL: diffuse large B-cell lymphoma. FFPE: formalin-fixed paraffin embedded; PD: progressive disease; PDTX: patient derived tumor xenograft.

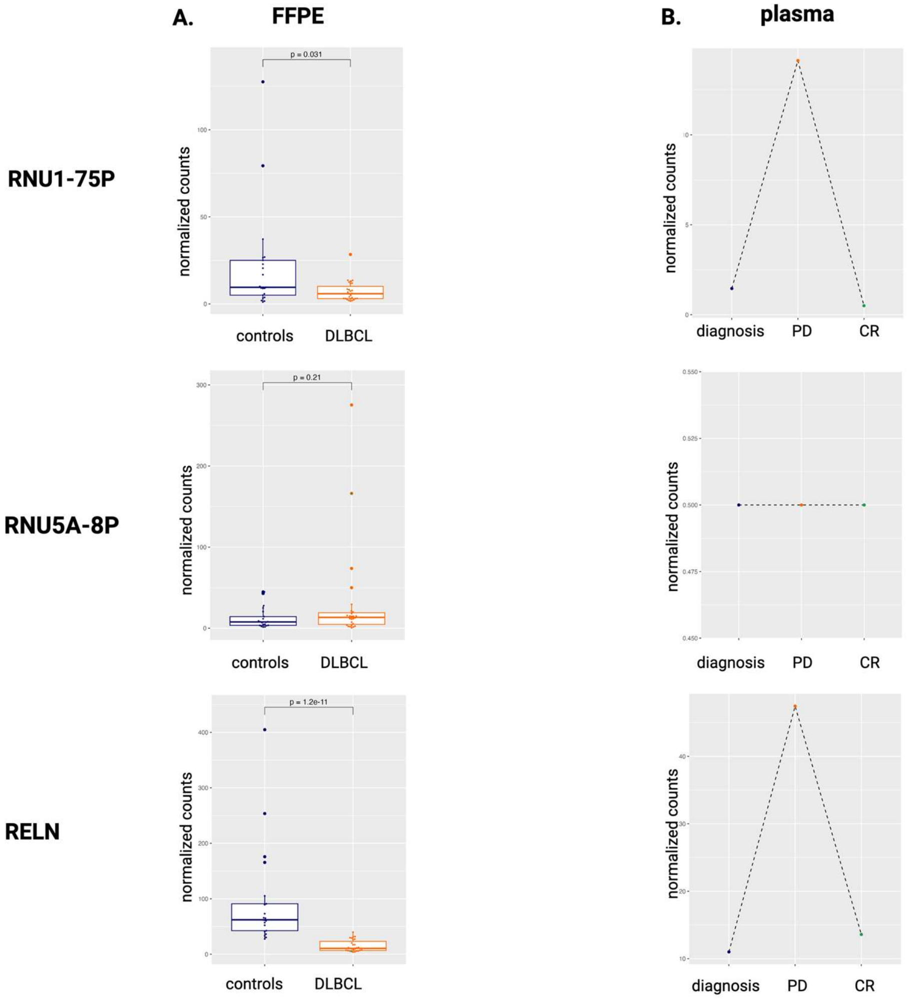

Supplementary Figure S10 (part 2): Normalized counts of the genes included in the signature between the FFPE tissue of DLBCL patients (n=27) and healthy controls (n=21). The DLBCL patient from whom the PDTX is derived is depicted in green. (A) Longitudinal abundance of these genes within the blood plasma of the patient from whom the PDTX is derived (B). CR: complete remission; DLBCL: diffuse large B-cell lymphoma. FFPE: formalin-fixed paraffin embedded; PD: progressive disease; PDTX: patient derived tumor xenograft.

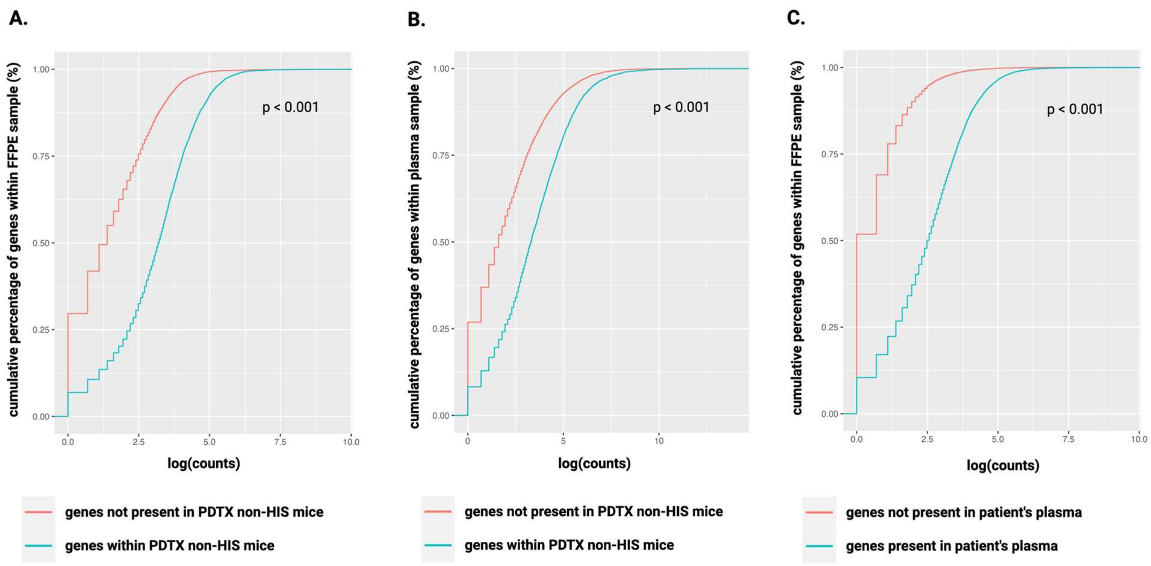

Supplementary Figure S11: Cumulative abundance of genes present (green) or absent (red) in the cfRNA of non-HIS PDTX PBS-treated samples within the diagnostic FFPE (A) and plasma (B) sample of the patient from which the xenograft is derived (two-sample Kolmogorov–Smirnov test, P < 0.001). Cumulative abundance of genes present (green) or absent (red) in the cfRNA of the patient's plasma within the matched diagnostic FFPE sample (C) of the patient from which the xenograft is derived (two-sample Kolmogorov–Smirnov test, P < 0.001). CfRNA: cell-free RNA; FFPE: formalin-fixed paraffin-embedded; HIS: human immune system; PDTX: patient derived tumor xenograft.

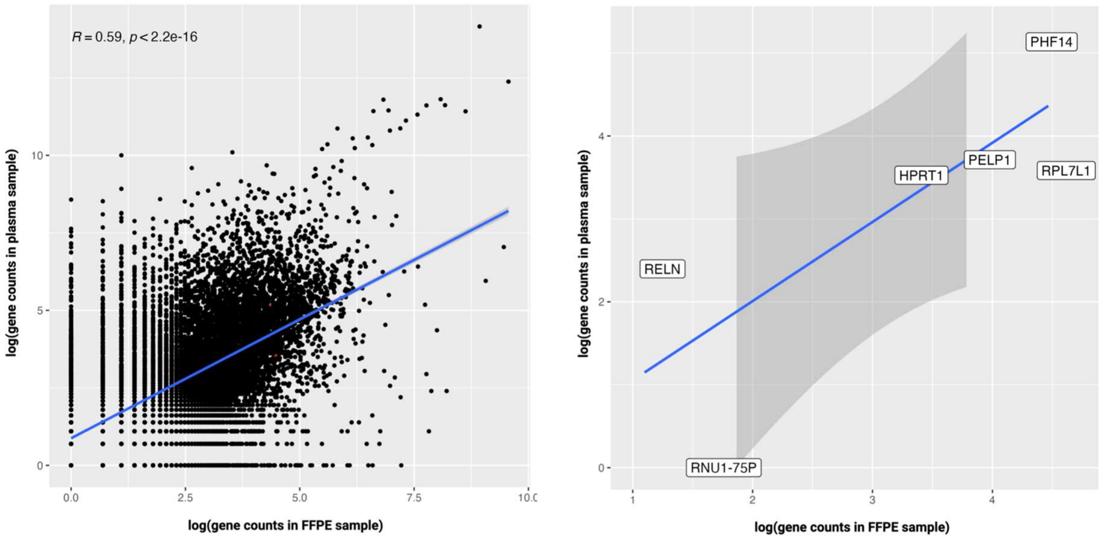

Supplementary Figure S12: Spearman correlation between the abundances of all genes in the diagnostic FFPE compared to the plasma sample of the patient from whom the PDTX is derived (left) and for the genes included in the tumor-specific signature of the PDTX model (right). FFPE: formalin-fixed paraffin-embedded; PDTX: patient-derived tumor xenograft; R: spearman correlation coefficient.

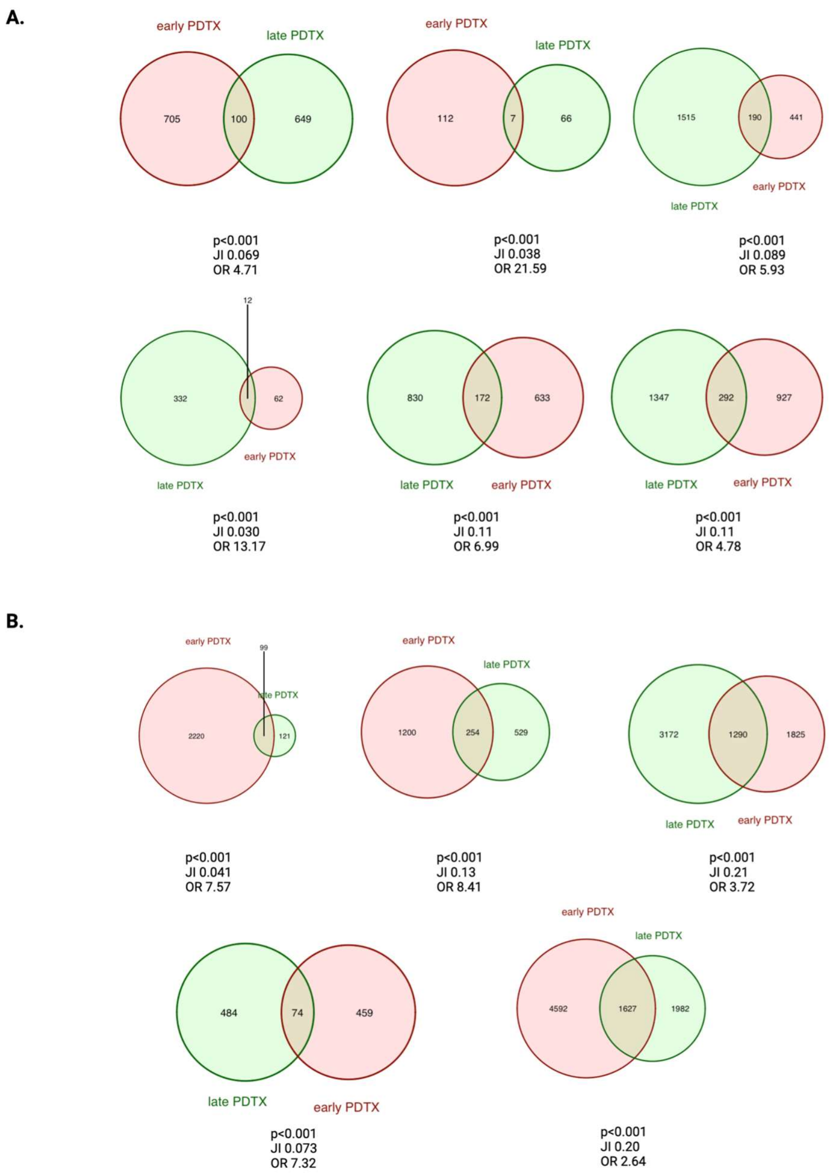

Supplementary Figure S13: Human gene overlap analysis between matched early and late stage PDTX longitudinal samples derived from the same PBS-treated non-HIS (A) and HIS (B) NSG mouse. HIS: human immune system; JI: Jaccard Index; NSG: NOD-*scid* IL2Rgnull; OR: odds ratio; PDTX: patient-derived tumor xenograft.

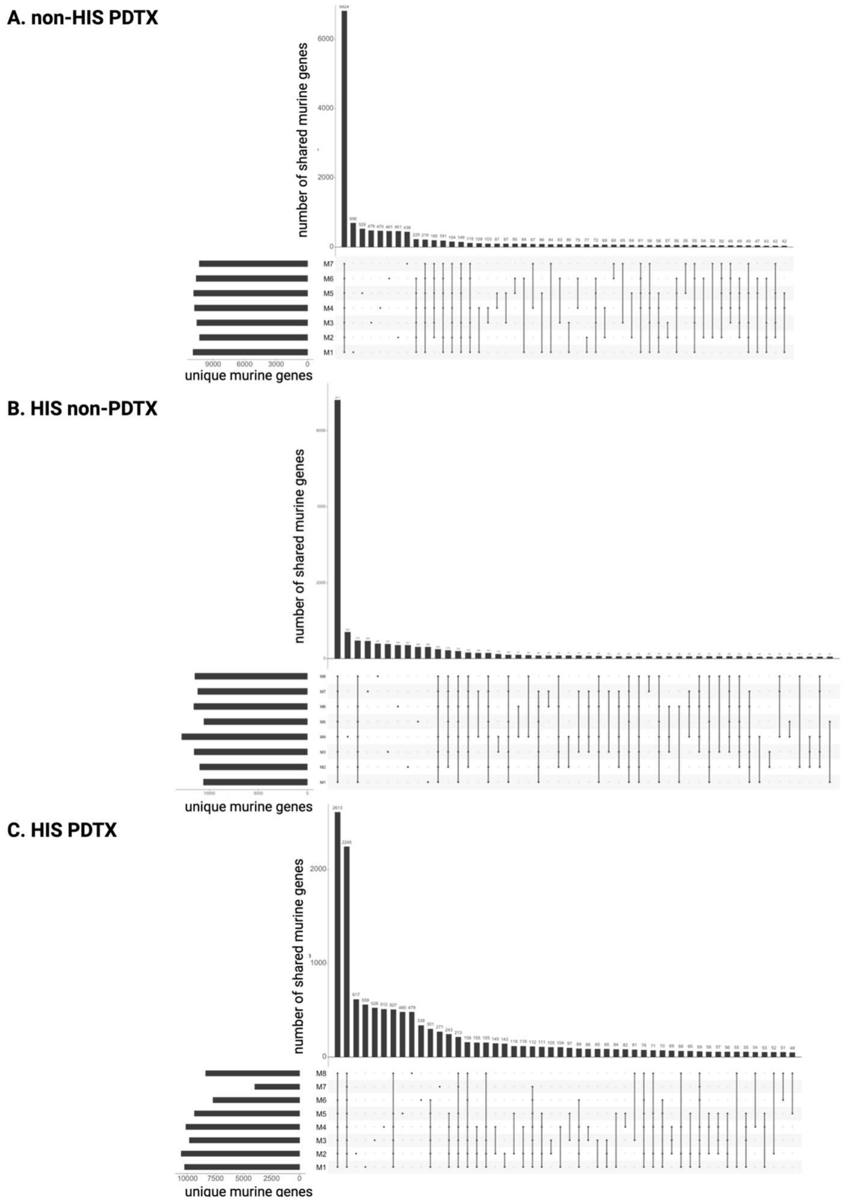

Supplementary Figure S14: Overlap analysis of unique murine genes among mice of the non-HIS PDTX (A), HIS non-PDTX (B), and HIS PDTX groups (C). For each group, the top section of each panel shows the number of unique genes for each mouse or combination of mice (indicated below), the lower left section depicts the number of unique murine genes per mouse, and the lower right section indicates which mice (as indicated by the dots) share unique genes. HIS: humanized; PDTX: patient derived tumor xenograft.

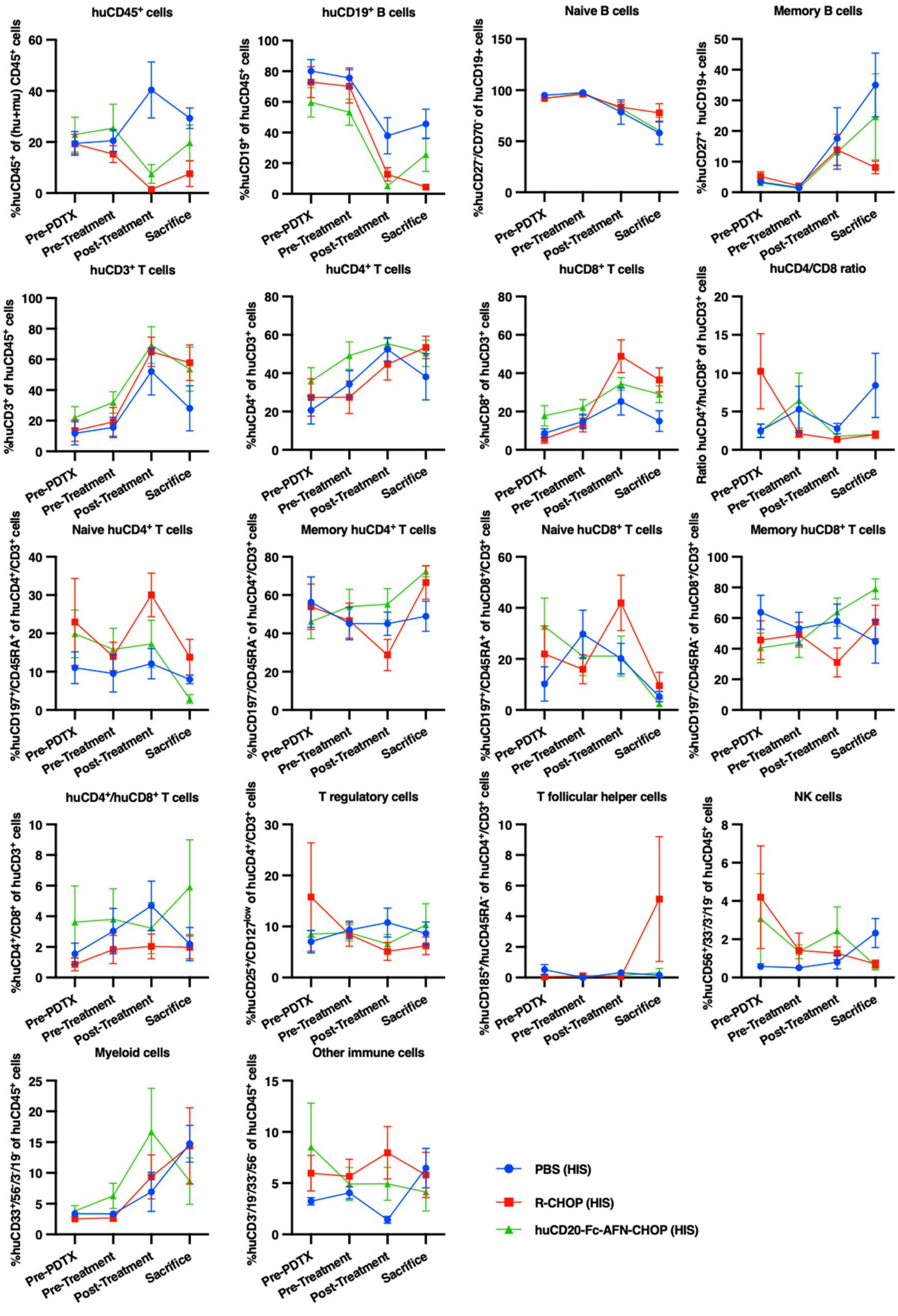

Supplementary Figure S15: Flowcytometric evaluation of blood at different timepoints in HIS NSG mice. HIS: human immune system; NSG: NOD-*scid* IL2Rgnull.

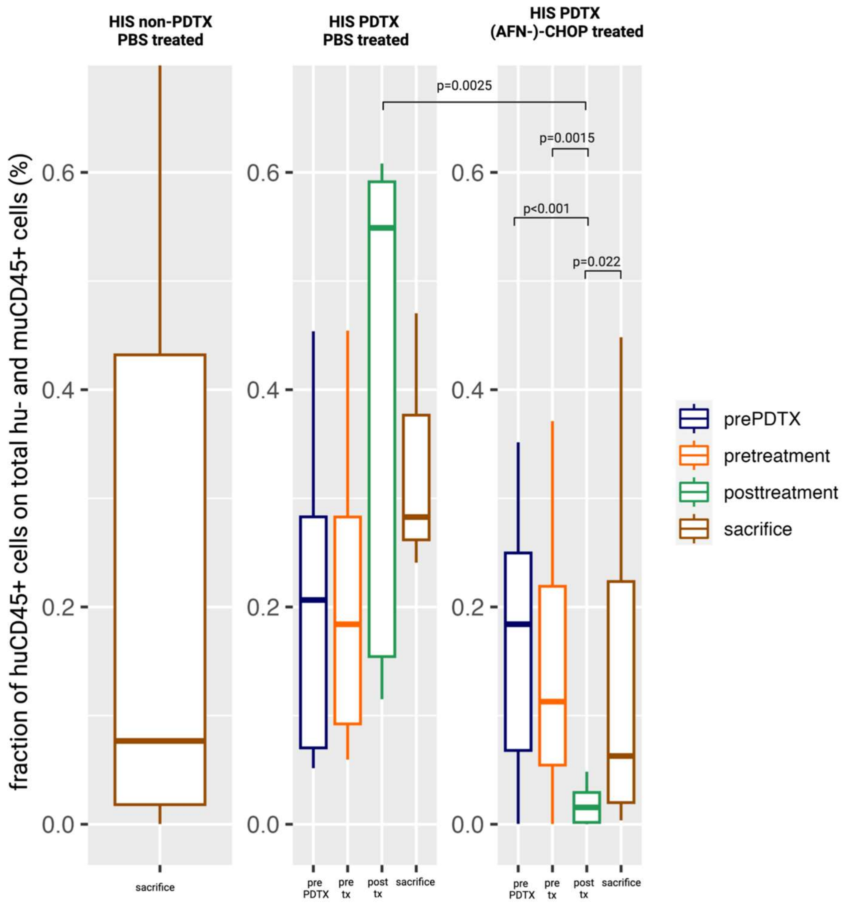

Supplementary Figure S16: The fraction of huCD45+ cells on total hu- and muCD45+ positive cells at different timepoints during xenograft implantation and treatment as determined using flowcytometric evaluation of blood. Significant p-values are shown. huCD45+: human CD45+ cells; muCD45+: murine CD45+ cells; PDTX: patient-derived tumor xenograft; pre-tx: pretreatment; post-tx:posttreatment.

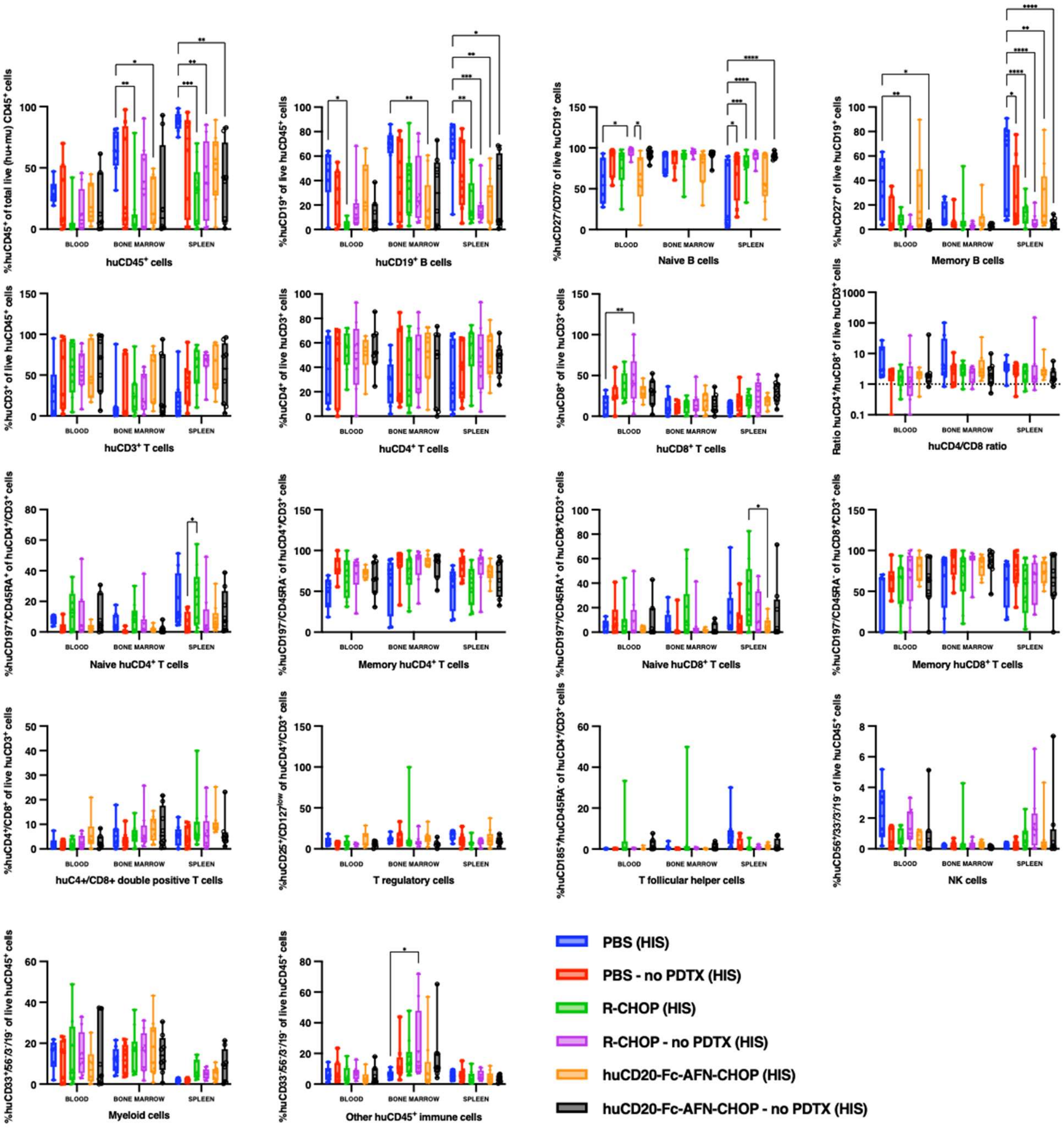

Supplementary Figure S17: Flowcytometric evaluation of blood, bone marrow and spleen at sacrifice. The panels show the differences in immune cell composition by organ, and by treatment at sacrifice timepoint.
*=P≤0.05;**=P≤0.01;***=P≤0.001; ;****=P≤0.<0001. HIS: human immune system; PDTX: patient-derived tumor xenograft.

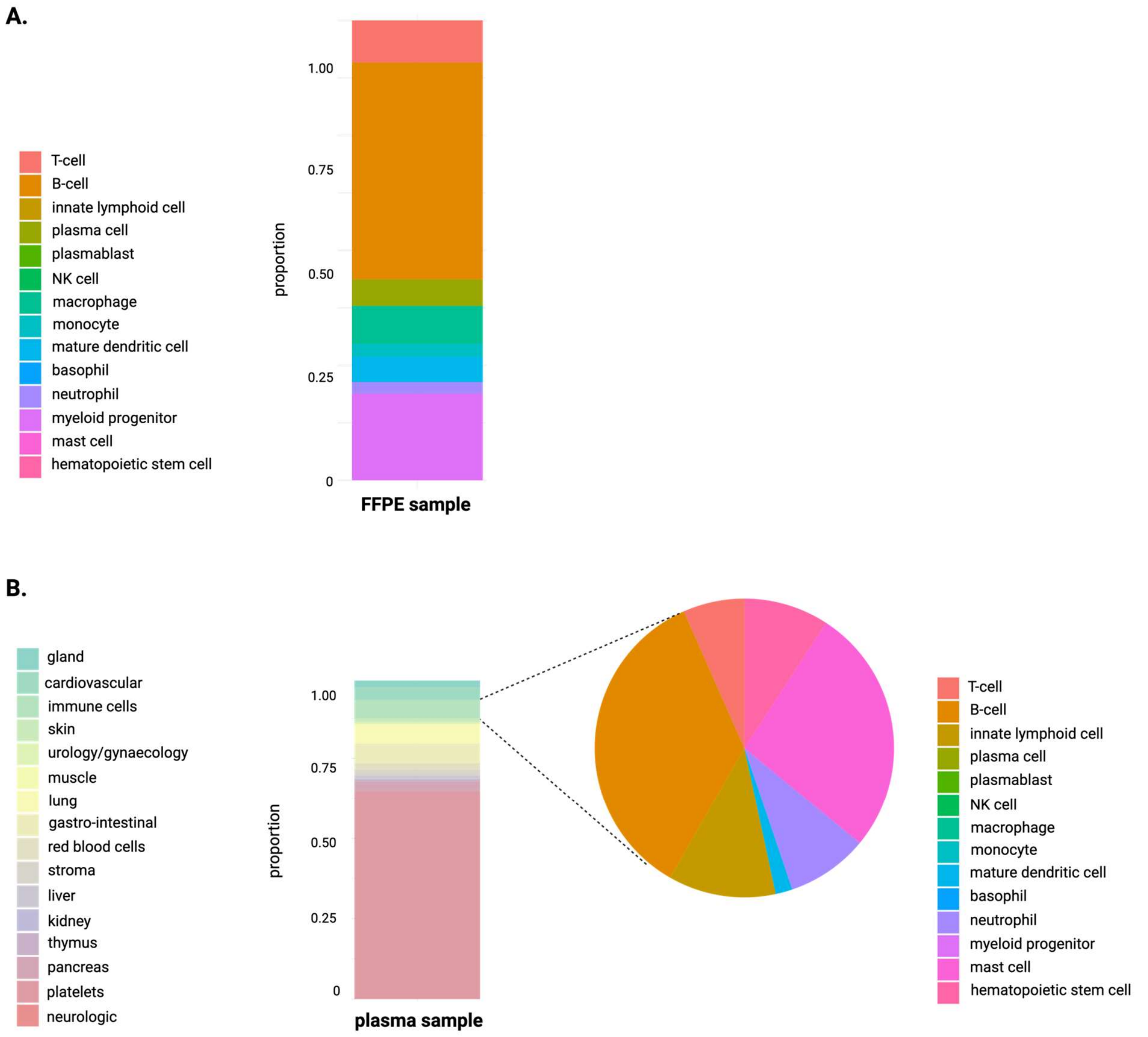

Supplementary Figure S18: Computational bulk deconvolution results on human cfRNA derived from the diagnostic FFPE (A) and blood plasma sample (B) of the patient from which the PDTX was derived. CfRNA: cell-free RNA; FFPE: formalin-fixed paraffin-embedded; PDTX: patient-derived tumor xenograft.

DLBCL patients	
**clinical characteristics**	
sex (m/v)	
male	11/27 (40.7%)
female	16/27 (59.3%)
age at diagnosis (yr)	65.04 (+/- 14.87)
Ann Arbor stage	
I	4/27 (14.8%)
II	7/27 (25.9%)
III	3/27 (11.1%)
IV	13/27 (48.1%)
bulky disease	7/27 (25.9%)
NCCN-IPI score	
low (0-1)	0/27 (0%)
low-intermediate (2-3)	14/27 (51.9%)
high-intermediate (4-5)	9/27 (33.3%)
high (>=6)	4/27 (14.8%)
comorbidities at diagnosis	9/27 (33.3%)
**tumor characteristics**	
COO: GCB	12/27 (44.4%)
COO: non-GCB	15/27 (55.6%)
DEL	12/27 (44.4%)
MYC expression	17/27 (63.0%)
BCL2 expression	18/27 (66.7%)
MYC rearrangement	2/27 (7.4%)
BCL2 rearrangement	6/27 (22.2%)
**first-line treatment**	
R-(mini)CHOP	27/27 (100%)
**laboratory values**	
mean LDH (IU/L)	374.59 (+/-288.36)
mean ß2 microglobulin (mg/l)	2.51 (+/- 0.90)
mean igG (g/l)	10.86 (+/- 6.10)
**outcome after first-line therapy**	
CR	17/27 (63.0%)
R/R	10/27 (37.0%)
**outcome of R/R patients after second- and third-line treatments**	
CR	3/10 (30.0%)
death	7/10 (70.0%)
**outcome after any therapy**	
CR	20/27 (74.1%)
death	7/27 (25.9%)

Supplementary Figure S19: Demographics, clinical characteristics, first-line treatments, and outcomes of DLBCL patients of which the diagnostic FFPE sample was included. Percentages and standard deviations are written in parentheses. COO: cell-of-origin; CR: complete remission; DEL: double-expressor lymphoma; DLBCL NOS: diffuse large B-cell lymphoma not otherwise specified, GCB: germinal center B-cell type; LDH: serum lactate dehydrogenase; NCCN-IPI National Comprehensive Cancer Network International Prognostic Index score; R/R: refractory/relapsed disease.
